# Efficacy of Probiotics as Prophylaxis for Urinary Tract Infections in Premenopausal Women: A Systematic Review and Meta-Analysis

**DOI:** 10.7759/cureus.18843

**Published:** 2021-10-17

**Authors:** Victor A Abdullatif, Roger L Sur, Eli Eshaghian, Kellie A Gaura, Benjamin Goldman, Pranav K Panchatsharam, Nathaniel J Williams, Joel E Abbott

**Affiliations:** 1 Research, Western University of Health Sciences, Pomona, USA; 2 Urology, UC San Diego Health, San Diego, USA; 3 Urology, Ascension Macomb-Oakland Hospital, Warren, USA; 4 Urology, Pacific West Urology, Las Vegas, USA

**Keywords:** uti prophylaxis, premenopausal, lactobacillus, recurrence, rutis, utis, urinary tract infections, probiotics

## Abstract

Introduction: Although antibiotic therapy has been the mainstay of prophylaxis and treatment of urinary tract infections (UTIs), antibacterial resistance has led to increased incidence of infections and healthcare spending in both community-acquired and nosocomial UTIs. This has led to an active exploration of alternative remedies for both the prophylaxis and treatment of UTIs, especially in women with recurrent urinary tract infections. Probiotic supplementation is one novel intervention that has been studied as a prophylactic measure in patients with UTIs. The current systematic review and meta-analysis was conducted to evaluate the efficacy of probiotics for prophylaxis in UTIs in premenopausal women.

Methods: Detailed search strategies for each electronic database were developed for PubMed, EMBASE, and Scopus to identify relevant literature published between 2001-2021. RevMan 5.3 statistical software was used to analyze data in studies. The random-effects model was used for pooling the data. The risk of bias and study quality were assessed using Cochrane Collaboration’s tool for assessing risk of bias in included studies. The scope of focus for this review was premenopausal adult women with a history of one or more UTI. The intervention consisted of a probiotic regimen for which the goal was to enhance the defensive microflora of the urogenital tract. Studies comparing a probiotic regimen to a placebo regimen were included. These studies’ primary outcome was the proportion of women with at least one symptomatic bacterial UTI in each group (i.e., UTI recurrence rate) in the 12-month period following probiotic intervention. This study extends the work of researchers who systematically investigated the scientific literature on probiotics in the prevention of urinary tract infections with a particular focus on premenopausal women.

Results: After screening, three parallel-group randomized-controlled trials (RCTs) were included. We estimated the overall pooled data of these three studies with a total of 284 participants to have met the predefined inclusion criteria and were therefore included in this review. The results demonstrated that probiotics did not have a significant effect in the prophylaxis of UTIs. (Risk Ratio (RR): 0.59 confidence interval (CI): 0.26, 1.33), Heterogeneity: Chi² = 6.63, df = 2 (*p* = 0.04); I² =70%, Test for overall effect: Z = 1.27 (*p* = 0.20).

Conclusions*:* Probiotics did not demonstrate a significant benefit in reducing UTI recurrence compared to placebo in premenopausal women. However, more conclusive data is needed to determine the effect that probiotics have on strengthening the urogenital microbial barrier against pathogenic bacteria and protecting against UTI recurrence.

## Introduction and background

Urinary tract infections (UTIs), defined as acute bacterial infections that occur when bacterial pathogens invade the urethra and ascend into the urinary system resulting in an inflammatory immune response, are the most common type of infections in women and elderly men. In contrast to bacteriuria, UTI is characterized by the presence of symptoms consistent with an inflammatory response [1,2]. The diagnostic definition of a UTI is a positive urine culture in the presence of associated symptoms [3]. These infections often result in dysuria, urinary frequency, urgency, hematuria, and/or incontinence. For some individuals, a UTI is a single occurrence that is self-limiting or managed with antibiotics, but other individuals are predisposed to recurrence and have repeat infections [4]. It is estimated that 50-60% of women will experience a UTI in their lifetime, and 20-30% of women will experience UTI recurrence [5-7]. Recurrent infections can result in extensive morbidity and lead to a substantial financial burden from high annual healthcare expenses related to diagnosis and treatment. Because untreated UTIs can lead to kidney infection and sepsis, it is imperative that UTIs are appropriately treated in a timely manner. 

Risk factors for UTI include urinary tract obstruction and stasis of urine, such as in benign prostatic hyperplasia/enlargement, urinary tract strictures, constipation, and neurogenic bladder [8]. These place patients at higher risk for recurrent or persistent infection [9]. Additionally, patients with comorbid conditions such as hyperglycemia, hypoestrogenism, pregnancy, nephrolithiasis, chronic or intermittent catheterization, anatomic abnormalities, and/or foreign bodies are more vulnerable to bacterial invasion of the urothelium [9]. The most frequent cause of UTI in premenopausal women is the transfer of host pathogens, commonly uropathogenic *Escherichia coli *from the rectum or vagina to the urethra following sexual intercourse.

Current behavioral modifications for UTI prophylaxis include maintaining adequate hydration, front-to-back wiping patterns, regular bowel movements, and post-coital voiding [10]. It is recommended that premenopausal women are administered a single-dose antibiotic after sexual intercourse if symptoms are associated with sexual activity. Patients are often encouraged to provide urine samples for culture when symptoms are present in order for physicians to obtain accurate antibiograms, as antibiotics are foundational to UTI management. In the majority of cases, antibiotics lead to complete disease remission. However, in patients with recurrent urinary tract infections (rUTIs), defined by the American Urology Association as two or more UTIs in a six-month period or three or more UTIs in a 12-month period, [11] patients may require multiple antibiotic prescriptions and/or extended antibiotic courses to achieve adequate treatment.

In patients with rUTIs, the current clinical approach for prophylaxis is a long-term, low-dose regimen of an antimicrobial agent, such as trimethoprim or nitrofurantoin [11]. Unfortunately, some bacteria have become resistant to the most frequently prescribed antibiotics which may result in UTI recurrence due to impaired clearance of said bacteria. This phenomenon has motivated the study of alternative non-antibiotic therapies, such as methenamine-hippurate, cranberry tablets, D-mannose, and probiotics, which are thought, in part, to counteract microbial resistance by reducing the need for antibiotic treatment [12]. 

The use of probiotics as a strategy in the management of patients with rUTIs is a relatively new field of study. It is largely based on research that has disproven the long-held notion that the human bladder is a sterile environment as more research points to the existence of a genitourinary microbiome [13,14]. The literature has demonstrated that, similar to the gastrointestinal tract, the urogenital tract contains a variety of microbes that play a role in homeostasis of the urinary microenvironment by altering local metabolite concentrations and pH [14]. With the advent of next-generation sequencing and advanced culturing techniques, researchers have been able to identify the bacteria that make up the resident flora in the bladder [15]. Although a consensus on the exact makeup of the bladder microbiota has yet to be reached, research on urinary symbiotic and pathogenic microorganisms has found remarkable differences between healthy populations and those suffering from various diseases [15]. 

Differences in beneficial and uropathogenic microorganisms have been observed between healthy populations and those with frequent UTIs. Quantities of beneficial urinary microorganisms, such as *Lactobacillus* and *Bifidobacterium*, have been shown to be decreased in patients with predisposing risk factors for UTI. Particular species of lactobacilli (LB), namely, *Lactobacillus iners, Lactobacillus crispatus, *and* Lactobacillus jensenii,* have been found to predominate in healthy premenopausal women [16-18]. Moreover, intravaginal estrogen, a well-studied and effective form of non-antibiotic prophylaxis for UTI in postmenopausal women, has been shown to increase the concentrations of LB in the vagina [19]. The proposed mechanism is primarily through the acidification of the vaginal canal. LB are lactic acid-producing bacteria and their presence results in a decrease in vaginal pH. This decrease in vaginal pH thwarts the growth of microorganisms and has been found to have a protective effect against UTI recurrence [20]. More recent literature has added to this paradigm by revealing similarities in vaginal and bladder microbiota [14]. On the basis of these research findings in tandem, supplementation with microorganisms (probiotics) is theorized to promote colonization of protective microbes and play a role in UTI prophylaxis and the maintenance of urinary health. Thus, probiotics have been proposed as a non-pharmaceutical option for UTI prophylaxis. Multiple studies have evaluated the efficacy of these beneficial microorganisms in the context of UTI prophylaxis [21-23]. 

A Cochrane review published in 2015 reported low evidence of probiotics in preventing UTIs in adults and children [24]. In contrast to the Cochrane review, we excluded men and children from our analysis and chose to focus exclusively on premenopausal women. This decision was made for two reasons: 1) the risk profiles in men and children differ from those in premenopausal women; 2) the incidence of UTIs is significantly higher in premenopausal women compared to men and children. Secondly, the Cochrane review included a study conducted by Czaja et al. in 2007 [25] which was the phase 1 trial of the subsequently completed Stapleton study [21]. The results of the Czaja study are therefore not estimable and were excluded from our analysis. Thirdly, in contrast to the Cochrane review, we excluded the Baerheim study from 1994 [26] for two reasons: 1) the outcome measure in the study was the total incidence of UTIs rather than recurrence rate in both arms, rendering the results as not estimable for relative risk reduction; 2) the authors' method for diagnosing UTI, which they defined as lower urinary tract symptoms (dysuria, urinary frequency, and/or suprapubic discomfort) and all of the following on urinalysis/urine culture: leukocyturia (≥ 5 leukocytes/HPF), bacteriuria (≥ 10^4^ colony-forming units [CFU]/mL) of uropathogens, or any amount of *Staphylococcus saprophyticus* [26], did not fit modern diagnostic criteria of UTI per American Urology Association guidelines. Lastly, our review includes a study performed by Koradia et al. in 2019 [22] that was not included in previous analyses. Our analysis includes data from this recent study to extend the work of researchers who systematically investigated the literature on probiotics in the prevention of urinary tract infections. To evaluate the efficacy of probiotic use as prophylaxis for UTI in premenopausal women, we performed a systematic review and meta-analysis of parallel-group randomized controlled trials (RCTs). We compared probiotics and placebo for UTI prophylaxis in premenopausal female patients with a history of at least one UTI. The primary outcome of interest was the proportion of subjects with one or more UTI in the 12 month period following probiotic intervention.

## Review

Methods

Information Sources and Search Strategy

The Cochrane Handbook for Systematic Reviews of Interventions [27] and the guidelines presented in the Preferred Reporting Items for Systematic Reviews and Meta-Analyses (PRISMA) statement [28] were used in the design of this systematic review and meta-analysis. Detailed search strategies for each electronic database were developed as described in Appendix 1. PubMed, EMBASE, and Scopus databases were searched for relevant reports from 2001-2021, and appropriate database-related search strategy modifications, such as the use of truncations, wildcards, and filters, were incorporated. We also scanned the references of all the included studies and used citation alerts to identify recent, updated publications and new studies. Search terms are located in Appendix 1. This meta-analysis is exempt from ethics approval as the data collected and synthesized is from previous clinical trials in which informed consent was already obtained by the individual trial investigators.

Inclusion and Exclusion Criteria

In order to properly evaluate the studies for inclusion and exclusion criteria, the reviewers (JEA, NJW) first chose to define the term “probiotic” as it is used in most current literature. In agreement with this definition, the term probiotics in our study refers to microorganisms that are either orally, rectally, or vaginally administered in a sufficient amount to alter the resident microbiota [29,30]. Therefore, only studies that utilized probiotics that satisfy this definition were included in our analysis. Parallel-group randomized controlled trials (RCTs) comparing probiotics with placebo for preventing UTIs between 2001 and 2021 were included. If the study randomized a population of premenopausal adult women with a history of one or more UTI within the 12 months before entering the study, the study was included in the analysis. One of the exclusion criteria was any intervention that utilized non-probiotic regimens. Interventions that aimed to alter the bladder microbiota via mechanisms that did not satisfy the aforementioned definition of “probiotics” were excluded. For example, several studies utilized a deliberate instillation of saline into the urethra/bladder (i.e., intravesical administration) with a particular strain of *Escherichia coli.* Although this intervention can be considered a probiotic, it fails to satisfy the contingency of probiotics as being orally, vaginally, or rectally administered microorganisms. Studies performed prior to 2001 were also excluded to maintain a focused analysis on the most current research surrounding the microbiome. Studies that exclusively evaluated the *treatment* of active UTIs, versus those evaluating *prophylaxis* as delineated in our inclusion criteria, were excluded. We did not exclude studies that evaluated both prophylaxis and treatment due to the significant recurrence rate of UTIs in premenopausal women. Finally, conference papers, abstracts, reviews, case reports/series, and retrospective studies were excluded from the analysis.

Studies Selection

**Figure 1:**
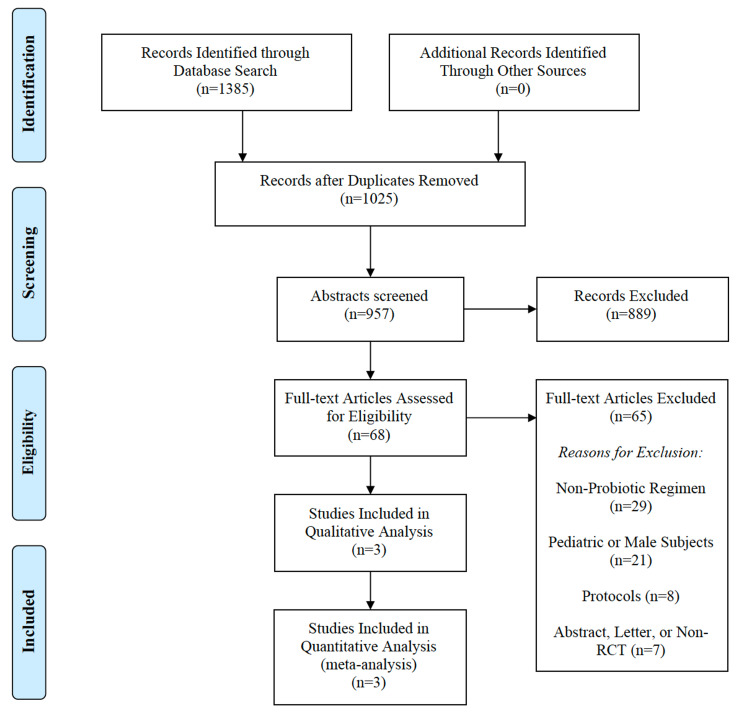
PRISMA Flow Diagram PRISMA - Preferred Reporting Items for Systematic Reviews and Meta-Analyses, RCT - randomized-controlled trial

The literature screening process for the selection of eligible studies was performed per PRISMA guidelines and is shown as a flow diagram in Figure 1. Two reviewers (JEA, NJW) screened the titles and abstracts of the articles retrieved through the electronic search and obtained the full-text articles for relevant studies. The kappa statistic was used to quantify the degree of agreement between the reviewers’ independent searches. The reference lists of relevant studies included keywords relating to probiotic supplementation and urinary tract infections. Both reviewers searched for RCTs that utilized probiotics as the independent variable and UTIs as the dependent variable. The relevant studies that met the inclusion criteria were assessed by all reviewers and retrieved for review.

Types of Intervention

The types of probiotic supplements included oral gelatin capsules, beverages, or vaginal suppositories. The bacterial strains and dosages included in the probiotic regimens were one of the following: 1) 100 ml of a beverage containing *Lactobacillus* GG at a dose of 4 x 10^10^ CFU/100 ml; 2) vaginal suppositories containing *Lactobacillus crispatus* CTV-05 (Lactin-V; Osel) at a dose of 5 x 10^8^ CFU per suppository; 3) capsules of BKPro-Cyan containing *Lactobacillus acidophilus* PXN 35 and *Lactobacillus plantarum* PXN 47 at a dose of >5 x 10^8^ CFU/capsule.

Types of Participants

Participants included premenopausal adult women with a past medical history of at least one UTI.

Types of Outcome Measures

The primary outcome in the included studies was the proportion of women with at least one symptomatic bacterial UTI in each group (i.e., UTI recurrence rate) in the 12-month period following probiotic intervention. The authors of the three included studies defined a UTI as one of the following: 1) symptoms (frequency, urgency, dysuria, hematuria, nocturia, fever or back or hip pain) and a positive urine culture with growth > 10^5^ CFU/mL upon presentation; 2) symptomatic uncomplicated cystitis defined as one or more typical UTI symptoms (dysuria, frequency or urgency) and pyuria and positive urine culture ≥ 10^2^ CFU/mL of one or more uropathogen (*E. coli *or other *Enterobacteriaceae*, *Enterococcus* species, or group B streptococci) or ≥ 10^5^ CFU/mL *Lactobacillus* as a single organism; 3) symptomatic uncomplicated cystitis defined as the presence of dysuria, frequency, urgency, suprapubic pain, and/or hematuria and > 10^3^ CFU/mL of uropathogens in a midstream sample of urine.

Study Quality Assessment and Risk of Bias Assessment

**Figure 2:**
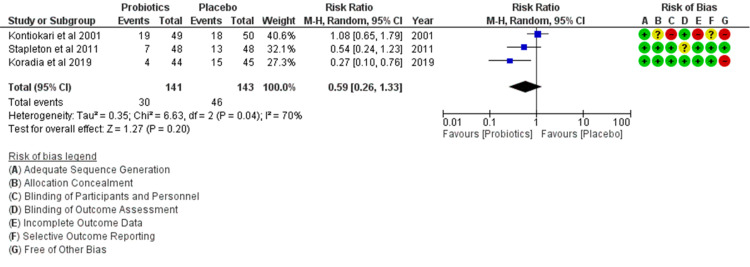
Forest plot with risk of bias summary of the included studies CI = Confidence Interval; Random = Random-Effects Model; M-H = Mantel-Haenszel [31]; DF = Degrees of Freedom; I2 = Heterogeneity Kontiokari et al. [23]; Stapleton et al. [21]; Koradia et al. [22]

All articles were evaluated according to the Cochrane Collaboration’s tool for assessing risk of bias (RoB) [27] to determine internal validity, and conflicting judgments were resolved by the reviewers. Two reviewers (JEA, NJW) independently performed the study quality assessment and any disagreements in assessment were resolved upon comparing the recorded data with a third author (VAA). Each trial was assessed for risk of bias associated with the method of sequence generation (selection bias), allocation concealment (selection bias), blinding of participants and personnel (performance bias), blinding of outcome assessment (detection bias), addressing incomplete outcome data (attrition bias), and outcome reporting. If a study's domain was deemed adequate, it was assigned a low likelihood of bias (green). If the study's domain was considered to be inadequate, it was assigned a high risk of bias (red). If it was equivocal whether the domain was adequate, risk of bias was deemed to be unclear (yellow). Using these metrics, results were entered and figures were generated with Cochrane's RevMan 5.3. The results of the reviewers’ risk of bias are shown vis-à-vis forest plot results in Figure 2.

**Figure 3:**
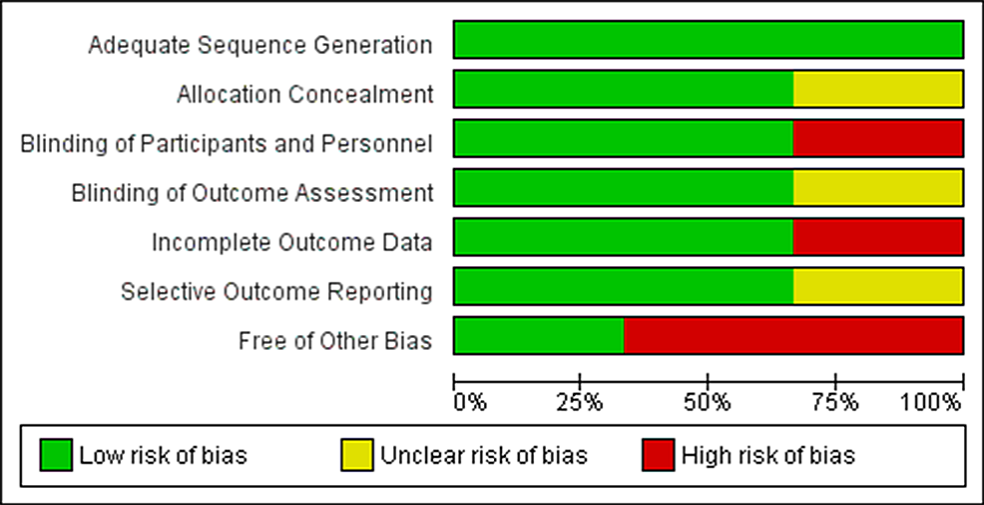
Risk of Bias Graph showing review authors' judgements about each risk of bias presented as percentages for all studies included in the analysis.

To control for omitted variable bias due to unobserved heterogeneity, a random-effects model was implemented in our bias assessment. All studies used random sequence generation for the selection of participants. Allocation concealment was used properly in all studies. Based on the reviewers' analysis, the overall risk of bias was low. Although the category of “other bias” was significantly higher than the rest (Figure 3), its effect was not significant enough to skew the overall percentage of bias in our assessment. Therefore, we determined that the included RCTs provided sufficient data for a meta-analysis of the primary outcome - namely, the rate of UTI recurrence after beginning prophylaxis with probiotics or placebo.

Data Extraction

Prior to data extraction, three authors (VAA, EE, KAG) created a pre-populated form with the variables for the analysis using Microsoft Excel. Data from all studies were extracted and compiled on Microsoft Excel by two reviewers (JEA, NJW) independently. These variables included study design, intervention type and dosing, study population, treatment duration/follow-up, outcome measures, and results.

Data Synthesis

Of the 284 patients which met the inclusion criteria of the study, participants were randomized into one of two groups: a treatment group and a control group. Participants in the treatment group were given a probiotic regimen and were reevaluated at various time points after intervention. The efficacy of the interventions for preventing UTI recurrence in the treatment group was compared with the effects of the placebo in the control group. Differences in risk ratios between probiotic prophylaxis (experimental) groups and placebo (control) groups were used to evaluate the efficacy of probiotics for prophylaxis against UTI recurrence.

Meta-Analysis Methodology

Risk ratios (RRs) with 95% confidence intervals (CIs) were calculated using per protocol analysis for every study. To combine the dichotomous outcomes of studies into a pooled RR, a random-effects model was used for the meta-analysis. Statistical heterogeneity among the studies was assessed using the I^2^ statistic, where I^2^ values of 25%, 50% and 75% indicated low, moderate, and high heterogeneity, respectively. Cochrane Collaboration’s RevMan version 5.3 was used to generate forest plots and perform the pooled analysis. Due to the presence of sparse data and the contingencies of the inclusion/criteria, the Mantel-Haenszel method was used to combine the risk ratios of the included studies.

Results

Study Selection

In our literature search, we identified a total of 1385 records. No additional records were identified from other sources. After removal of duplicates, 1025 records remained for review. After further review of the titles and abstracts of these records by two independent reviewers (JEA, NJW), 68 full-text articles were selected. We then excluded 65 studies that had one or more of the following characteristics: 1) pediatric or male subjects; 2) non-probiotic regimens; 3) non-randomized controlled protocols. Three studies with a total of 284 participants met the predefined inclusion criteria and were included in this review. A flow diagram of the screening process is shown in Figure 1. The details of each selected study are presented in Table 1.

**Table 1:** Details of the included studies RCT - randomized controlled trial; UTI - urinary tract infection; CFU - colony-forming units; Lactin V- *Lactobacillus crispatus*

Study	Study design	Study population	Sample size/Groups	Probiotic type/strain		Treatment duration and follow-up	Outcome measures	RR (95% CI), p value
Kontiokari et al 2001 [23]	Parallel RCT	Adult women with current UTI caused by *Escherichia coli*. Mean age (years): treatment group (32); control group 1 (30); control group 2 (29).	n=45 (treatment) n=50 (control)		*Lactobacillus GG *drink.	5 days/week for 1 year, 12 months of follow-up.	UTI recurrence rate.	1.11 (0.70-1.76), p = 0.8320.
Stapleton et al 2011 [21]	Parallel RCT	Premenopausal women with current, symptomatic, uncomplicated UTI. Median age (years): treatment group (21); control group (21).	n=48 (treatment) n=48 (control)		*L. crispatus* CTV-05 vaginal suppository (Lactin-V), 10^8^ CFU/mL.	Once daily for 5 days, 10 weeks of follow-up.	UTI recurrence rate.	0.54 (0.24-1.23), p = 0.2089
Koradia et al 2019 [22]	Parallel RCT (pilot)	Female subjects aged who had suffered ≥2 episodes of uncomplicated acute UTI in the last 6 months, or ≥3 episodes of uncomplicated acute UTI in the last 12 months. Median age (years): treatment group (34.6); control group (34.8).	n=44 (treatment) n=45 (control)		*Lactobacillus acidophilus* PXN 35 and *Lactobacillus plantarum* PXN 47 capsules (BKPro-Cyan), >5 x 10^8^ CFU/capsule.	Twice daily for 26 weeks, 3-month and followed up on days 45, 90, 135 and end-of-study.	UTI recurrence rate.	0.27 (0.10-0.76), p = 0.0053

Meta-Analysis of the Pooled Data

Heterogeneity was analyzed using a chi-squared test on N-1 degrees of freedom. An alpha value of 0.05 was used for statistical significance with the I^2^ test [32]. I^2^ values of 25%, 50% and 75% correspond to low, medium and high levels of heterogeneity, respectively. The rate of UTI recurrence was 21.3% (30/141) in the treatment group and 32.2% (46/143) in the control group. Our analysis of the data showed that probiotics did not have a statistically significant effect in reducing the rate of UTI recurrence (Risk Ratio (RR): 0.59 confidence interval (CI): 0.26, 1.33), Heterogeneity: Chi² = 6.63, df = 2 (p = 0.04); I² =70%, Test for overall effect: Z = 1.27 (p = 0.20) (Figure 2). Although the forest plot demonstrated a trend towards benefit in the treatment arms, there was no statistically significant difference in the combined analysis. This finding will be further discussed in the discussion section.

Publication Bias

Individual studies have an inherent risk of publication bias, which is defined as the tendency for authors of research to publish significant results. In other words, a study with a high association between the likelihood of a publication and the statistical significance of a study result is said to have a high publication bias [33]. Due to the small amount of included studies, an analysis of publication bias would likely be too underpowered to provide an accurate estimation [33]. Therefore, a funnel plot was not included in our meta analysis.

Discussion

The growing problem of antibiotic resistance has led researchers to investigate non-medical interventions for management of rUTIs. In the present study, we investigated the effect of probiotics on prophylaxis for UTIs in premenopausal women and found that probiotics do not have a significant effect. There has yet to be a consensus if probiotics are effective as prophylactics for UTI recurrence in premenopausal women. Insofar as probiotics have been studied as non-pharmaceutical options for UTIs, they have demonstrated minimal efficacy in both treatment and prophylaxis. It is not clear exactly which interventional variables (routes of administration, bacterial strains, dosages, or frequencies) are the most effective. This systematic review and meta-analysis serves to consolidate the literature in regards to premenopausal women. 

One mechanism that may explain the lack of protective effects of probiotics is the route of ingestion. A recent study conducted by Wolff et al. in 2019 found that an oral probiotic containing *Lactobacillus rhamnosus* GR-1 and *Lactobacillus reuteri* RC-14 (two strains of LB with theorized beneficial effects) did not significantly alter the ratio of uropathogens to lactobacillus (U/L ratio) [34]. This finding may explain the lack of benefit in the Kontiokari study in which the researchers used an oral beverage containing LB as the intervention. In contrast to the Kontiokari study, Koradia et al. demonstrated a significant risk reduction using a *Lactobacillus*-containing oral supplement. However, this probiotic supplement used in the Koradia study (BKPro-Cyan) contained cranberry proanthocyanidins which may have exerted an anti-inflammatory effect potentially impacting the study results. It has been suggested that phenolic compounds may have more general anti-inflammatory effects rather than any effects on bacterial adhesion in patients [35]. It remains unclear if a combination of probiotics and cranberry proanthocyanidins exerts a different effect than either one alone. 

In contrast to oral probiotic supplementation, Uehara et al. have demonstrated that *Lactobacillus* vaginal suppositories containing *L. crispatus* GAI 98332 significantly altered the vaginal microbiota [36]. There appears to be both increased colonization and viability of these beneficial microorganisms with vaginal administration when compared to oral. Reid et al. have documented cases in which enterococcal species were displaced by a simple intravaginal installation of *Lactobacillus rhamnosus* GR-1 and *Lactobacillus reuteri* RC-14 and resolved active UTI symptoms [37]. In the Kontiokari study, researchers utilized vaginal suppositories containing *Lactobacillus crispatus* CTV-05 and demonstrated increased LB colonization and a minor risk reduction against UTI recurrence in the treatment arm [23]. However, their findings were not statistically significant when considering differences in the risk ratios between the experimental and control groups in the study. Although it remains unclear as to what the ideal intervention is for UTI prophylaxis, intravaginal appears to be a more promising route of administration providing a more potent effect on microbe viability and colonization. Future research should be aimed at utilizing protocols with evidence of vaginal and urogenital colonization in both premenopausal and postmenopausal women.

Another variable that may have contributed to the conflicting evidence surrounding probiotics is the quality of the probiotics used in the studies. Some researchers rely on probiotic supplements from third-party websites. Wolff et al., the authors who found that oral LB did not significantly alter the ratio of uropathogens to LB, purchased a probiotic supplement from a third-party website (iherb.com) [34], which may have impacted their results. Recent literature suggests that there is a significant amount of variability in strain viability, dosing, contamination, and misidentification of bacterial strains between different probiotic supplements [38]. Without quality control and regulatory oversight in the supplement industry, products may lack clinically effective dosages. This is especially true in supplements that offer so-called “proprietary blends” in which exact amounts of all ingredients are not listed on labels. Therefore, the use of over-the-counter probiotics limits researchers from providing clinically valid results. Fortunately, most studies that involve the use of microorganisms as interventions implement advanced laboratory equipment to properly identify strains and quantify the concentrations of said strains. However, quality control is one metric that should be implemented in all studies involving probiotic interventions. Although probiotics are generally recognized as safe [39] with minimal to low likelihood that misidentification is likely to cause significant harm, patients seeking to benefit their health from these supplements may be misled and incur significant costs. Another complicating factor is the variability in the microbiome makeup between individuals [40], which suggests that there is no one-size-fits-all approach to probiotic supplementation. Before clinicians can make evidence-based recommendations for probiotic supplementation, advanced technology may be required to identify the makeup of individual microbiota and determine if adequate colonization is occurring upon administration.

Another variable that may partially explain the result of our review is the inclusion of only adult premenopausal women (i.e., 18 to 50). The literature on rUTIs in premenopausal women suggests that the risk factors in this age group differ from those in the postmenopausal age group [41]. Premenopausal women are more likely to have recent antibiotic use, an individual history of UTIs, use of an intrauterine device, use of spermicidal condoms, and recent sexual intercourse [6]. These risk factors have an indeterminate level of impact on the ratio of uropathogens to protective microorganisms in this population and are not entirely controlled for in most published studies. Perhaps more importantly is the composition of the female bladder microbiota pre- and post-menopause. Antonio et al. have demonstrated that postmenopausal women have 25% to 30% of the LB in the vagina compared to premenopausal women [16]. Other researchers have also demonstrated that estrogen therapy may increase this percentage of vaginal LB to 60-100% [42]. Because premenopausal women do not appear to be as deficient in beneficial LB, our impression is that premenopausal women may not experience as much benefit from supplemental probiotics containing LB. The findings of Uehara et al. support this notion as they have demonstrated a significant reduction in the number of UTI recurrences in postmenopausal women treated with intravaginal probiotics [36]. Along with implementing standardized bacterial strains, future researchers may aim to administer probiotics via routes that have proven to effectively colonize the vagina, bladder, and urogenital tract. 

The reviewers considered lag time as a potentially compromising variable in the results. In the Stapleton study, subjects were treated for acute UTI at visit 1 with standard therapy, given probiotics seven to 10 days after visit 1, and then reevaluated at 10 weeks [21]. It is unclear which antibiotic the authors deemed “standard therapy.” It was also unclear how long the subjects were treated for. If the antibiotic regimen was long enough to affect probiotic microbe viability, this may have prevented adequate colonization of the protective microbes in the urogenital tract. This is another variable in methodology that requires standardization across studies wishing to assess the efficacy of probiotics in treatment/prophylaxis of UTIs. Our impression is that the follow-up methodology of the Koradia study for follow-up was ideal as subjects were reevaluated on days 45, 90, 135 and end-of-study [22]. This time frame allows for antibiotics to effectively clear uropathogens while also allowing for probiotic colonization to take place, insofar as oral supplements are able to do so.

During the review phase of our study, the authors noted novel literature on the deliberate establishment of asymptomatic bacteriuria with a particular strain of *Escherichia coli* having a significant effect in reducing the rate of reported UTIs in patients with incomplete bladder emptying. This intervention has been termed “bacterial interference,” in which the bladder is intentionally colonized with low virulence bacteria. Interestingly, RCTs that have evaluated this intervention as a strategy for UTI prophylaxis have found bladder instillation to be highly effective, particularly in patients with neurogenic bladder. In a literature review conducted by Falcou et al., researchers found that bladder interference with non-pathogenic strains of *Escherichia coli* (HU 2117 and 83972) was associated with statistically significant reductions in symptomatic urinary tract infections in patients with neurogenic bladder [43]. These findings suggest that bladder instillations may be the most effective way to colonize the bladder with beneficial microbes and reduce the colonization of uropathogens. Future researchers may choose to use this intervention in both neurogenic and non-neurogenic populations.

Strengths and Limitations

One strength of our study is the use of a research question that is narrow in scope. Because of strict adherence to the inclusion and exclusion criteria, we were able to effectively identify and synthesize data that directly related to the population of interest. The authors and reviewers were parsimonious about defining the population of interest, the interventions, and outcome measures (e.g., treatment v. prophylaxis). Because our focus was prophylaxis, we ensured that only studies evaluating prophylaxis were included. Although our criteria came with the cost of fewer studies, we believe that a strict adherence to said criteria leads to more definitive and clinically relevant conclusions. 

One limitation of the current study is the presence of interventional heterogeneity among the three included studies. The researchers of the included papers did not use identical probiotics (dose, strain, frequency, etc). Because of a lack in standardization of dose, strain, and frequency, evidence for the absence of a relationship between the probiotic interventions and outcomes cannot be definitively determined. This interventional variety may impact the quantitative analysis of the results. Although efforts were made to synthesize data that was as similar as possible with our inclusion criteria, no two studies were identical in their protocols. However, this discovery has allowed us to uncover a gap in the literature and the need for more RCTs implementing more standardized protocols.

## Conclusions

In this review, probiotics did not demonstrate a significant benefit in reducing UTI recurrence compared to placebo. The study that reported a statistically significant benefit may have been subject to confounding bias due to the presence of cranberry extract in the probiotic supplement. Despite our findings, it remains inconclusive as to what role oral and intravaginal probiotics have in UTI prophylaxis in premenopausal women. Deliberate bladder instillation with particular microbes is an interesting and promising intervention that should undergo additional study. These novel interventions designed to restore the urogenital microbiome may one day play a role in protecting individuals against various diseases, including functional diseases, bladder cancer, and inflammatory conditions and warrant further investigation.

Future trials should implement standardized routes of administration that have been shown to sufficiently enhance the quantity of protective microorganisms in the bladder. More conclusive data is needed regarding the effect probiotics have on strengthening the urogenital microbial barrier against pathogenic bacteria and protecting against UTI recurrence. This would require further exploration with long-term, multicenter RCTs with large sample sizes and standardized strains, dosages, routes, and outcome measures.

## Appendices

Appendix 1

Search terms

PubMed For searches conducted in the PubMed database, the following terms were used: ((“urinary tract infection”[Mesh] OR “urinary tract infection”[TIAB] OR “urinary tract infection therapy”[TIAB] OR “urinary tract infections prevention and control”[TIAB] OR “cystitis”[TIAB] OR “pyelonephritis”[TIAB] OR “bacteriuria”[TIAB] OR “pyuria”[TIAB] OR “pyuric “[TIAB] OR “pyurias”[TIAB] OR “utis “[TIAB] OR “uti”[TIAB])) AND (“probiotic agent”[Mesh] OR “probiotic agent”[TIAB] OR “probiotics”[TIAB] OR “symbiotic”[TIAB] OR “lactobacill*”[TIAB] OR “Yakult”[TIAB] OR “actimel”[TIAB] OR “proviva”[TIAB] OR “cultura”[TIAB] OR “verum”[TIAB] OR “bifidobacterium”[TIAB] OR “activia”[TIAB] OR “enterococcus”[TIAB] OR “streptococcus thermophiles”[TIAB] OR “saccharomyces”[TIAB] OR “diarsafe”[TIAB]).

EMBASE For searches conducted in EMBASE, the following terms were used: 1. ‘probiotic agent’/exp OR ‘probiotic agent’ OR probiotics:au OR synbiotic:jt OR lactobacilli$ OR ‘yakult’/exp OR yakult OR ‘actimel’/exp OR actimel OR ‘proviva’/exp OR proviva OR cultura OR verum OR ‘bifidobacterium’/exp OR bifidobacterium OR ‘activia’/exp OR activia OR‘enterococcus’/exp OR enterococcus OR ‘streptococcus thermophilus’/exp OR ‘streptococcus thermophilus’ OR ‘saccharomyces’/exp OR saccharomyces OR diarsafe; 2. ‘urinary tract infection’/exp OR ‘urinary tract infection’ OR (urinary:au AND tract:au AND infection:au AND therapy:au) OR ‘urinary tract infections prevention and control’:jt OR ‘cystitis’/exp OR cystitis OR ‘pyelonephritis’/exp OR pyelonephritis OR ‘bacteriuria’/exp OR bacteriuria OR ‘pyuria’/exp OR pyuria OR pyuric OR pyurias OR utis OR uti; 3. #1 AND #2.

Scopus For searches conducted in the Scopus database, the following terms were used: 1. TITLE-ABS-KEY (probiotic agent) OR TITLE-ABS-KEY (probiotics) OR TITLE-ABS-KEY (probiotic) OR TITLE-ABS-KEY (symbiotic) OR TITLE-ABS-KEY (lactobacillus) OR TITLE-ABS-KEY (yakult) OR TITLE-ABS-KEY (actimel) OR TITLE-ABS-KEY (proviva) OR TITLE-ABS-KEY (saccharomyces)); 2. TITLE-ABS-KEY (urinary tract infection) OR TITLE-ABS-KEY (urinary tract infection therapy) OR TITLE-ABS-KEY (cystitis) OR TITLE-ABS-KEY (pyelonephritis) OR TITLE-ABS-KEY (bacteriuria) OR TITLE-ABS-KEY (pyuria) OR TITLE-ABS-KEY (pyuric) OR TITLE-ABS-KEY (pyurias) OR TITLE-ABS-KEY (uti)); 3. #1 AND #2.
